# Magnetic resonance imaging related anxiety and workflow: impact of a child-friendly audio-visual intervention

**DOI:** 10.1007/s00247-025-06308-0

**Published:** 2025-07-04

**Authors:** Sanae van der Vleuten-Chraibi, Sanne Nauts, Dobromiła Barańska, Emilio J. Inarejos Clemente, Jonas Sterup Bovin, Nadia Najafi, Julian A. Luetkens, Marianne Alison, Hilla M. Biermann, Fabian Peckman, Privender Saini

**Affiliations:** 1 Philips Medical Systems, Veenpluis 6, 5684 PC Best, Netherlands; 2 Department of Radiology, The Polish Mother’s Memorial Hospital Research Institute, Lodz, Poland; 3 Department of Diagnostic Imaging, Sant Joan de Deu Hospital, Barcelona, Spain; 4 Department of Radiology, Herlev Hospital, Herlev, Denmark; 5 Department of Anesthesiology and Perioperative Medicine, University Hospital of Brussels (UZBrussel), Vrije Universiteit Brussel (VUB), Brussel, Belgium; 6 Department of Diagnostic and Interventional Radiology, University Hospital Bonn, Bonn, Germany; 7 Department of Pediatric Imaging, AP-HP, Hôpital Robert Debré, Université Paris Cité, Inserm, NeuroDiderot, Paris, France

**Keywords:** Anxiety, Audio-visual content, Awake-scanning, Magnetic resonance imaging, Patient experience, Pediatric, Workflow

## Abstract

**Background:**

Magnetic resonance imaging (MRI) is the diagnostic imaging modality of choice for pediatric patients. However, it can be challenging to scan young children awake while maintaining a high image quality with an efficient, patient-friendly workflow.

**Objective:**

We investigated if an audio-visual intervention with specially designed pediatric content could reduce MRI-related patient anxiety and workflow-issues in children during an awake MRI.

**Materials and methods:**

In six European hospitals, children (*n* = 175; aged 6–12 years) were scanned with child-friendly content (intervention) or without (control). Staff recorded children’s stress levels before, during, and after the MRI on a 6-point Likert scale. Scan issues (i.e., repeat scans and interruptions) were recorded by staff and extracted from the MRI logfiles.

**Results:**

The stress level of young children (aged 6–10 years) in the intervention group decreased more strongly from before to after the MRI compared to the control group, *F*_(2,96)_ = 7.84, *P* < 0.001. They also had significantly fewer scan issues as reported by staff, *F*_(1,169)_ = 8.36, *P* = 0.004, Cohen’s *d* = 0.58, and as logged by the MRI system, *F*_(1,156)_ = 8.10, *P* = 0.005, Cohen’s *d* = 0.45. The used pediatric content showed no significant effects on older children (aged 10 + years).

**Conclusion:**

A child-friendly audio-visual intervention can help reduce stress levels of young children (aged 6–10 years) and support a smooth workflow.

**Trial registration number:**

NCT05089955, date: 2021–10-22.

**Graphical Abstract:**

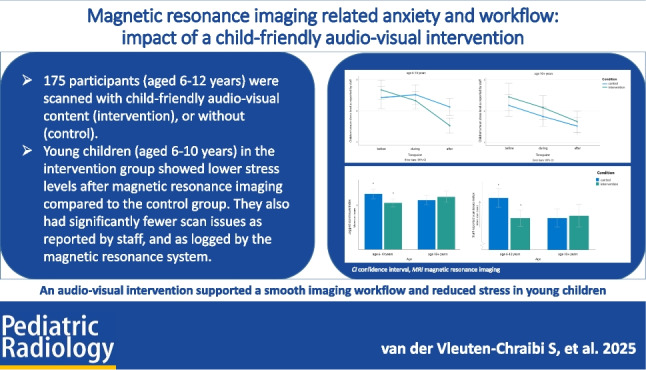

**Supplementary Information:**

The online version contains supplementary material available at 10.1007/s00247-025-06308-0.

## Introduction

Magnetic resonance imaging (MRI) is the imaging modality of choice in children to achieve accurate diagnosis, with its excellent soft tissue-contrast and absence of ionizing radiation. However, for young children, undergoing an MRI can be a difficult experience. Young children are often scanned using sedation or anesthesia to ensure an efficient workflow that yields high-quality diagnostic images. Scanning children awake has several advantages, such as shorter waiting lists [1], less time spent in the hospital [2], lower cost [2, 3], a reduced risk of unexpected incidents [4–6], and reduced exposure to potentially neurotoxic anesthetic agents [7–9].

When children are scanned awake, providing optimal support for stress management is important, as high levels of anxiety in a medical setting can contribute to the development of pediatric medical traumatic stress [10, 11]. Various interventions exist to support awake scanning of young children [12–15], e.g., using patient preparation with mock scanners, virtual reality, or play therapy [16–21]. Some hospitals use audio-visual systems that enable children to watch a movie or play a game during their MRI [22–25], sometimes combined with patient preparation [1, 26, 27]. Such interventions benefit children and their families and can be cost-effective for hospitals [18, 26, 28].

In the present study, we investigated the effectiveness of an audio-visual intervention with pediatric content. Pediatric patients (aged 6–12 years) were scanned with audio-visual intervention (intervention group) or without (control group). We measured children’s situational anxiety levels (general feelings of stress, worry, nervousness, or unease) and the incidence of scan-related issues (e.g., repeat sequences, lengthy pauses between sequences, failure to obtain diagnostic images).

## Materials and methods

This prospective study had a two-armed mixed methods design and ran between January 2023 and May 2024. Ethical/institutional review board (IRB) approval or exemption was obtained by all hospitals. Before participating, parents/guardians provided written informed consent; child assent was obtained according to local regulations.

### Participants

Patients were recruited at six European hospitals, located in Poland, Spain, Denmark, Germany, Belgium, and France.

Inclusion criteria were as follows: aged 6–12 years; scheduled for a first head-first, awake MRI; child and parent/guardian speak the local language. Exclusion criteria were: neurological disorders affecting the ability to lie still; developmental/cognitive disorders.

The study was designed to include 300 patients. Due to recruitment challenges and delays in acquiring IRB approvals, the study was closed after including 175 patients.

### Materials

**Fig. 1:**
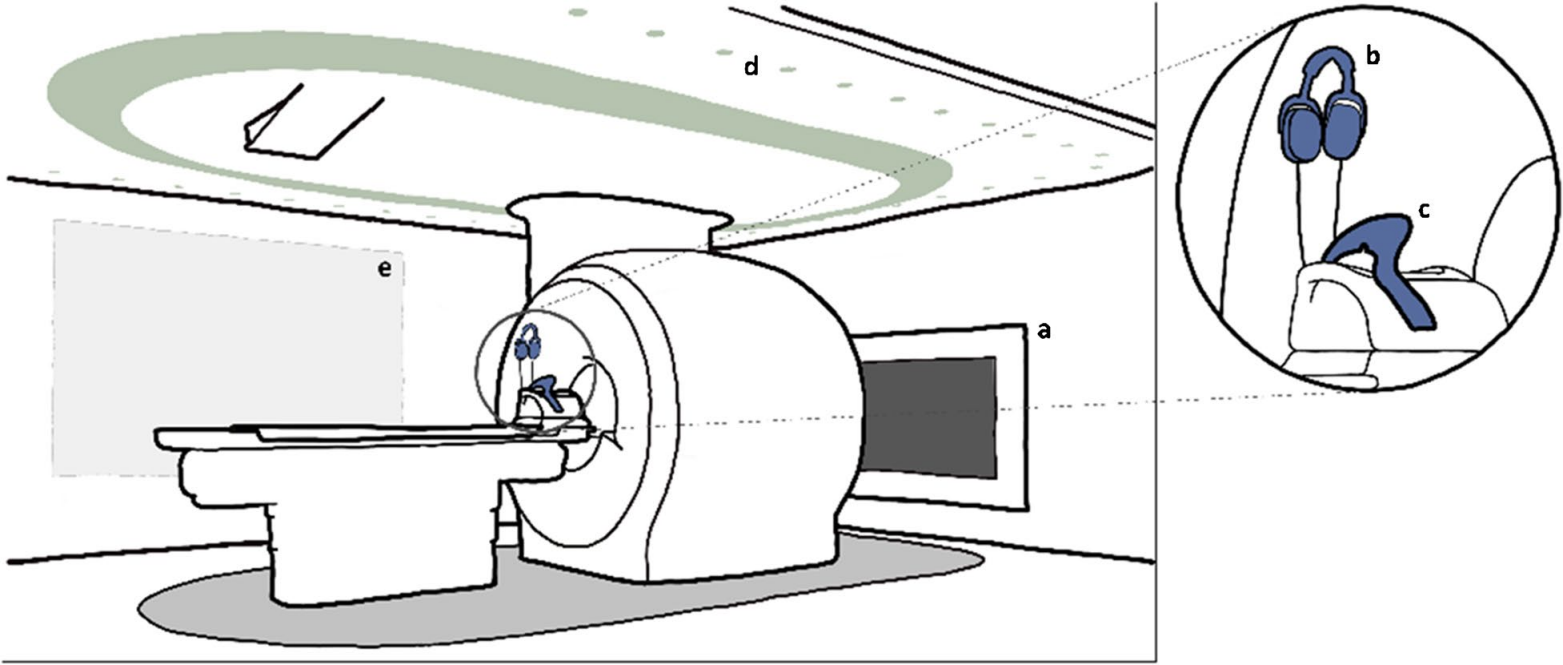
Magnetic resonance imaging room equipped to provide child-friendly audio-visual intervention, including an in-bore screen (**a**), headphones (**b**), mirror (**c**), colored lighting (**d**), and wall projection (**e**)

All patients were scanned on Philips MRI systems equipped with Ambient Experience (Philips Medical Systems, Best, the Netherlands), which consists of an in-bore screen, colored lighting using ceiling-mounted fixtures, and a sound system (Fig. 1), and in one hospital, an additional projection on the wall. The setup was designed to create a calming atmosphere and provide guidance and positive distraction during the scan.

**Fig. 2:**
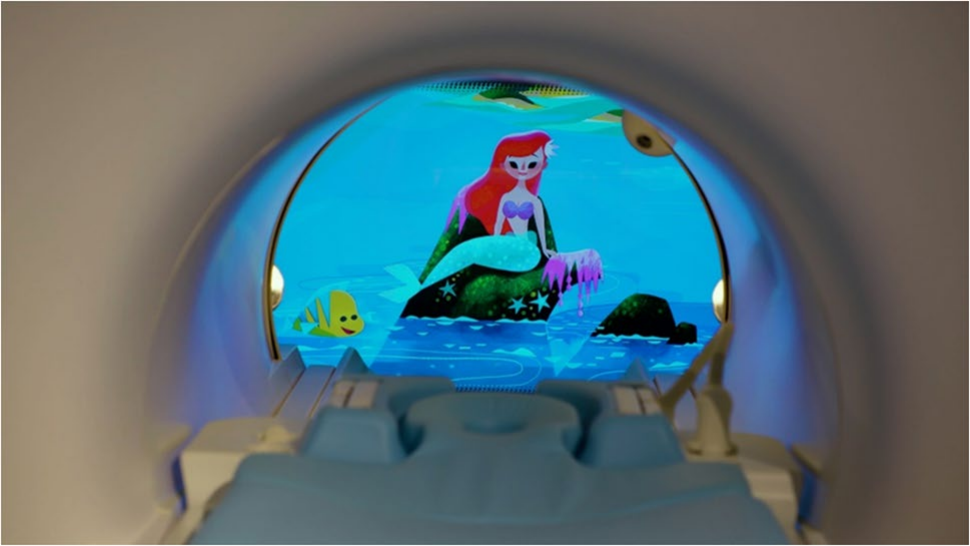
Child-friendly content displayed on the in-bore screen of the audio-visual system

In collaboration with The Walt Disney Company Limited (TWDC), five calming 5-min clips were created (TWDC, Burbank, CA). To provide comfort to children through familiarity, the clips featured numerous well-known animated TWDC characters such as Mickey Mouse, Ariel, Winnie the Pooh, Spider-Man, and Yoda (Fig. 2). The content created has gentle, slow-paced visuals because fast-paced content can reduce young children’s ability to follow instructions or regulate their behavior [29–32]. Since head motion and eye movements during an MRI may cause motion artifacts, character movement was focused on center-screen. Pre-made sets of clips were created, varying from 15–25 min, and displayed on the interface, showing the first clip’s character image. Control was provided to improve patient calmness [11, 33] by allowing children to choose the first clip to watch.

### Measures

Data were collected using questionnaires for patients and staff, and by extracting data from MRI system logfiles (i.e., exam length, number of repeat scan sequences, number of pauses between sequences). All questionnaires are presented to patients and staff in local languages. We either used validated translations or validated the items by having them translated by a professional translator and reviewed by bilingual experts in behavioral science and clinical application. Staff of all sites (three to five individuals per site), including radiographers, (assistant) radiologists, and research coordinators, were trained using the same training set to ensure the questionnaires were administered consistently. In the current paper, we report the results for the primary outcome regarding patients’ MRI-related situational anxiety (feelings of stress, worry, nervousness, or unease), and for measures related to workflow (scan issues). The supplemental materials present other measures that were included in the study.

To measure stress, staff answered the question “How stressed was the child?” on a scale from 1 *(not stressed at all)* to 6 *(extremely stressed)* at three time points: before, during, and after the MRI. Staff also filled out the modified Yale preoperative anxiety scale [34, 35] before and during the MRI. This is an observational scale on which staff rates four different types of behavior that children may show in a medical setting (e.g., crying, clinging to a parent). Supported by staff, patients answered the question “Can you tell us how you are feeling about your MRI exam?” on a 6-point visual analogue scale from 1 *(*v*ery relaxed)* to 6 *(very tense)* before and after the MRI. Child-appropriate equivalents of the labels were presented in the local language.

Scan issues were measured in two ways: as reported by staff and as extracted from the MRI logfiles. Five items (see Table 1; *α* = 0.85) were converted into z-scores and averaged to form an aggregated *staff-reported scan issues* index. Principal component analysis confirms a one-factor solution for these items. The resulting staff-reported scan issues index violates assumptions with regard to normal distribution; therefore, we (1) added a constant to the values (+ 2), eliminating negative values; and (2) transformed the resulting index using a reciprocal transformation (1/*x*). Even after transformation, the distribution is not fully normal, but the shape of the distribution is improved, as well as the values for Kolmogorov–Smirnov (*D*) and Shapiro–Wilk (*W*) tests; before transformation *D* = 0.22 and *W* = 0.80; after transformation *D* = 0.13 and *W* = 0.96.

**Table 1:** Items used to create a staff-reported scan issues-index

Label	Item	Scale
Repeat sequences	How many sequences did you have to repeat for this patient?	Numeric, open-ended
Number of pauses	How many times did you have to intervene or pause the scan procedure because the child was not cooperative?	Numeric, open-ended
Scan duration	Please estimate the duration of the MRI examination compared to how long a similar MRI examination normally takes	1 (shorter than normal) to 6 (longer than normal)
Diagnostic images	Were you able to obtain diagnostic images?	1 (yes), 2 (yes, but)^a^, 3 (no)
Image quality	Please estimate the quality of the final MRI images. Please only consider the image quality due to patient movement	1 (very high) to 6 (very low)

*MRI* magnetic resonance imaging

^a^The response scale for “diagnostic images” included multiple options (“yes, but did apply sedation”; “yes, but accepted images of suboptimal quality”; “yes, but had to contact radiologist”), which were aggregated into “yes, but…”

From the MRI-logfiles, three items (see Table 2; *α*=0.81) were converted into *z*-scores and averaged to form an aggregated *logged scan issues* index. Principal component analysis confirms a one-factor solution for these items. The resulting logged scan issues-index violated assumptions with regards to normal distribution; therefore, values are again transformed, (1) adding a constant (+2); and (2) using a reciprocal transformation (1/*x*) on the resulting index. After transformation, normality is confirmed.

**Table 2:** Items used to create a logged scan issues-index

Label	Definition
Pause duration	The sum of all pause durations between scan sequences
Repeat sequences	Number of duplicate sequence names during one exam
Pauses > 30 s	Number of pauses exceeding 30 s

### Procedure

**Fig. 3:**
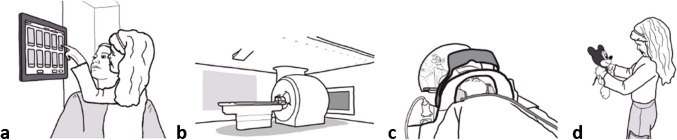
Procedure (intervention group). **a** Patient selects pediatric content on the interface. **b** Content is displayed in the scan room using ceiling lighting, in-bore display*,* and optionally wall projection. **c** During the scan, distraction and guidance are visible for the patient in a head-mounted mirror. **d** After the scan*,* the child receives a soft toy

In the waiting or dressing room, prior to the introduction of the intervention, patients filled out the *before* questionnaires. When entering the MRI room, patients in the intervention group selected TWDC content (pediatric content), which was displayed on a screen behind the bore, visible in a head-mounted mirror (Fig. 3), accompanied by sound and light. Patients in the control condition had their MRI without audio-visual intervention (without pediatric content) and did not receive audio-visual distraction and guidance. All patients received staff guidance or reassurance when needed. After the MRI, all patients filled out the *after* questionnaire and received a stuffed toy to thank them for participating in the study. Staff filled out questionnaires before, during, and after the MRI.

After enrollment, patients were assigned to the intervention or control group using block randomization. Patient assignment to a condition was based on scanning day and did not consider equal age distribution within the group. Each hospital applied a consistent block randomization schedule suiting their workflow, i.e., switching conditions after blocks of 12–13 patients, every five patients, or switching conditions every week. By grouping patients of the same condition sequentially, turning the audio-visual intervention on/off had minimal impact on staff workflow.

### Statistical analysis

Data were analyzed using IBM SPSS statistics for Windows (version 29, IBM Corp., Armonk, NY). To ascertain the effect of the audio-visual intervention on staff-reported stress, we conducted a 2 (age: 6–10, 10 +) × 2 (condition: intervention, control) × 3 (timepoint: before, during, after) repeated measures analysis of variance (ANOVA) with Bonferroni correction for multiple comparisons. For staff-observed anxiety (modified Yale preoperative anxiety scale) and child-reported anxiety, we conducted a 2 (age) × 2 (condition) × 2 (timepoint) repeated measures ANOVA with Bonferroni correction. To ascertain the effect of the audio-visual intervention on staff-reported scan issues, we conducted a 2 (age) × 2 (condition) ANOVA. For the scan issues from the MR logfiles, we conducted a 2 (age) × 2 (condition) analysis of covariance (ANCOVA) with the number of MRI sequences as a covariate, since the number of repeat sequences and pauses was limited by the total number of sequences.

## Results

In total, 175 children (89 girls, 84 boys, two unrecorded) participated in the study; the number of patients enrolled per hospital varied (range [4,55]). Most children (78%) had a head/brain MRI, and one in three children (34%) required an intravenous injection for contrast. A total of 60% of the patients had never had an MRI before. As shown in Table 3, older children (aged 10 + years) were underrepresented in the intervention condition.

**Table 3:** Patients per age group per condition

	**Control** ***n* = 90**	**Intervention** ***n* = 85**	**Overall** ***n* = 175**	***P***
Age 6–10 years Age 10 + years	44 46	56 29	100 75	0.032

Data were collected in three age categories. Early termination of the study led to some smaller cell sizes (e.g., 17 children aged 6–8 years in the control group). The two lower age categories (6–8 and 8–10 years) are aggregated into one category (6–10 years) to ensure sufficiently large cell sizes for proper analyses

Data from the MRI logs were available for 161 children; within this group, average exam time was 27.42 min (standard deviation = 8.01, range [14,53]).

**Fig. 4:**
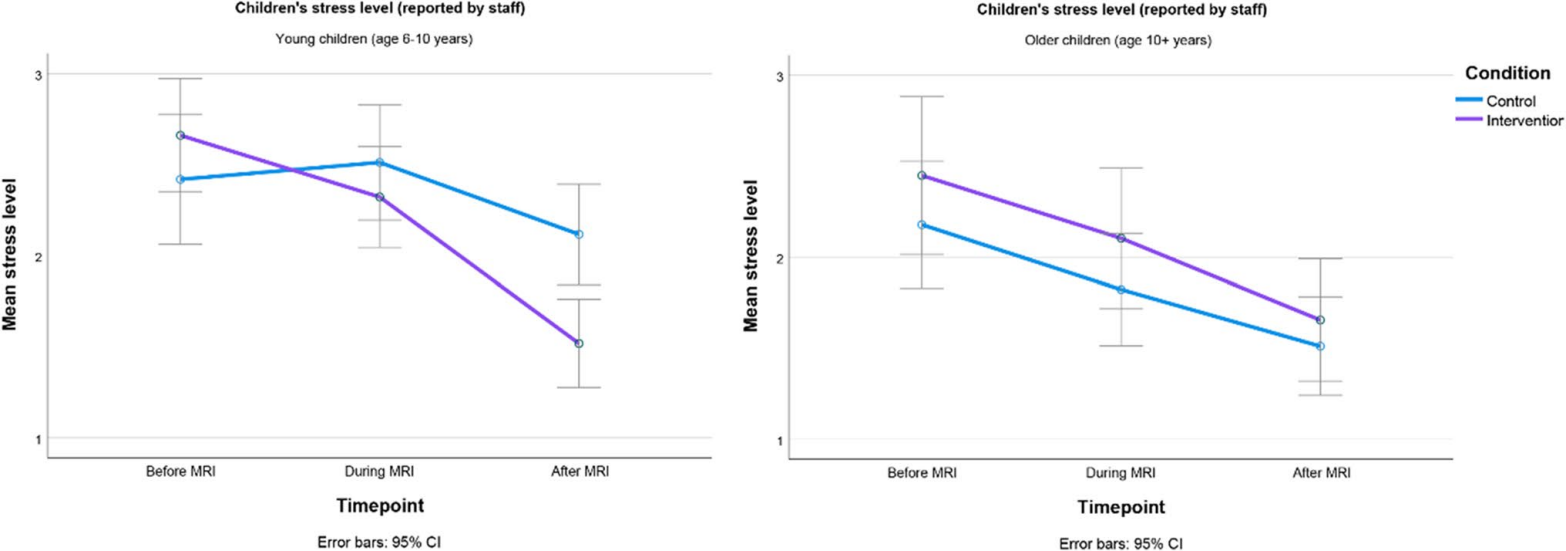
Stress levels (as reported by staff) across timepoints, for both conditions and age groups. *CI* confidence interval, *MRI* magnetic resonance imaging

Results of the staff-reported stress are presented in Table 4 and Fig. 4. The measure showed a main effect of timepoint: *F*_(2,168)_ = 36.40, *P* < 0.001, indicating that stress levels peaked before the MRI, became lower during the exam, and were at their lowest point after the exam. There was no significant main effect of condition, and there were no significant interactions between condition and age, or between timepoint and age (*F*s < 1.64; *P*s > 0.20). There was a significant effect of age and a significant interaction between timepoint and condition (*F*s > 4.96, *P*s < 0.03), but these were qualified by a marginally significant three-way interaction between timepoint, age, and condition *F*(2,333) = 2.53, *P* = 0.08.

To explore the three-way interaction in detail, we conducted separate 2 × 3 (condition*timepoint) ANOVAs for the two age groups. For children aged 10 + years, there was no significant interaction between timepoint and condition (*F* < 1). For children aged 6–10 years, there was a significant interaction between timepoint and condition *F*(2,194) = 7.84, *P* < 0.001, suggesting that the intervention resulted in a stronger decrease of stress from before to after the MRI (cf. the control condition; see Table 4 and Fig. 4). After the MRI, young children in the intervention condition were significantly less stressed than young children in the control condition, *P* = 0.004, Cohen’s *d* = 0.584 [36] (Table 4). In line with our expectations, the audio-visual intervention significantly reduced staff-reported stress, but only for children aged 6–10 years.

**Table 4:** Stress levels for the control and intervention condition, as reported by staff before, during, and after magnetic resonance imaging

	**Patients: age 6–10 years**			**Patients: age 10 + years**		
	**Control** ***M (SD)***	**Intervention** ***M (SD)***	***P***	**Control** ***M (SD)***	**Intervention** ***M (SD)***	***P***
Before MRI	2.42 *(1.12)*	2.66 *(1.24)*	0.25	2.18 *(1.17)*	2.45 *(1.18)*	0.29
During MRI	2.51 *(1.28)*	2.32 *(1.03)*	0.46	1.82 *(0.81)*	2.10 *(1.05)*	0.27
After MRI	2.12^a^ *(1.26)*	1.52^a^ *(0.76)*	0.004	1.51 *(0.76)*	1.66 *(0.81)*	0.51

*M* mean, *MRI* magnetic resonance imaging, *SD* standard deviation

^a^Significantly different at *P* < 0.05

Contrary to our expectations, for staff-observed anxiety (modified Yale preoperative anxiety scale) and child-reported anxiety, there were no significant main effects of condition (intervention, control), and no significant interactions with condition (*F*s < 1). Although staff-reported stress significantly decreased for young children in the intervention condition (cf. the control condition), we did not find such an effect on anxiety levels as reported by children, or those observed by staff using the modified Yale preoperative anxiety scale.

For staff-reported scan issues, there was a significant interaction between age group and condition, *F*_(1,169)_ = 4.54, *P* = 0.034. For children aged 6–10 years (but not for children aged 10 + years), the audio-visual intervention significantly reduced staff-reported scan issues, *F*_(1,169)_ = 8.36, *P* = 0.004, Cohen’s *d* = 0.58 (Table 5).

**Table 5:** Staff-reported scan issues index for the control and intervention condition

Patient age: 6–10 years			Patient age: 10 + years		
Control *M (SD)*	**Intervention** ***M (SD)***	***P***	**Control** **M *(SD)***	**Intervention** ***M (SD)***	***P***
0.46^a^ *(0.18)*	0.36^a^ *(0.15)*	0.004	0.36 *(0.16)*	0.38 *(0.18)*	0.74

*M* mean, *SD* standard deviation

^a^Significantly different at *P* < 0.05

For logged scan issues, there was a significant interaction between age and condition, *F*_(1,156)_ = 6.63, *P* = 0.011. For children aged 6–10 years (but not for children aged 10 + years), the audio-visual intervention significantly reduced logged scan issues, *F*_(1,156)_ = 8.10, *P* = 0.005, Cohen’s *d* = 0.45 (Table 6).

**Table 6:** Logged scan issues index for the control and intervention condition

Patients age: 6–10 years			Patient age: 10 + years		
Control *M (SD)*	**Intervention** ***M (SD)***	***P***	**Control** ***M (SD)***	**Intervention** ***M (SD)***	***P***
0.44^a^ *(0.18)*	0.36^a^ * (0.17)*	0.005	0.39 *(0.17)*	0.43 *(0.20)*	0.35

*M* mean, *SD* standard deviation

For interpretation ease, means of the reverse-coded index are presented, such that higher means signify more scan issues

^a^Significantly different at *P* < 0.05

## Discussion

Scanning young patients awake benefits children, their families, and healthcare systems. However, it can be difficult for healthcare providers to minimize patient stress and maintain an efficient workflow that yields high-quality images when children are scanned without sedation or anesthesia. We investigated if an audio-visual intervention with child-friendly content can alleviate children’s anxiety and reduce scan issues, such as repeat scans and scan interruptions. In collaboration with The Walt Disney Company, we developed MRI-specific content with calming music and lighting, featuring familiar characters that children know and trust.

The current study only included patients scheduled for an awake scan, therefore the results do not include potential benefits for patients referred for a MRI with sedation, nor for patients with neurological/cognitive disorders.

Our findings suggest that the audio-visual intervention significantly reduced scan issues for younger children (aged 6–10 years), but not for older children (aged 10 + years). This effect holds regardless of whether scan issues are reported by staff or measured directly in the MRI log files. Next to reducing scan issues, the audio-visual intervention significantly reduced stress levels in younger children (aged 6–10 years), as rated by staff. Younger children’s stress levels decreased to levels comparable to those of older children (age 10 + years), who experienced low stress levels regardless of condition. Although we found effects on children’s stress levels as reported by staff, we unexpectedly did not find a significant reduction in behavioral indicators of anxiety (modified yale preoperative anxiety scale) or child-reported anxiety. These measures showed limited variance and may have lacked sensitivity which, combined with low statistical power, may account for the unexpected absence of statistically significant effects. The modified Yale preoperative anxiety scale, for example, has mostly been validated in pre-surgery contexts, with very young children [34], and may have lacked sensitivity in older children and non-surgery contexts (where behaviors such as crying or clinging to a parent may be less common).

The intervention did not significantly affect stress levels or the incidence of scan issues for older children (10 + years), who showed low stress levels and for whom scan issues were rare. While it seems likely that younger children stand to benefit most from audio-visual interventions, in the current study, the content of the clips may have been more engaging for younger children. Given that the clips were too short to cover all MRIs fully, children likely watched some content multiple times: something that may be particularly bothersome for older children. Moreover, given that only 29 children aged 10 + years were included in the intervention-condition, it is possible that we did not find a significant effect of condition for this group due to a lack of statistical power. Future research should employ longer clips that are more engaging for older children (10 + years) and include more children to ensure adequate statistical power.

Overall, the current research indicates that providing children with a calming environment and child-friendly in-bore content can reduce stress levels (as reported by staff) in young children (aged 6–10 years). Moreover, it can support clinicians by reducing scan issues and disruptions, thereby improving the workflow and facilitating clinicians to achieve high-quality diagnostic images. While the study was not focused on anesthesia reduction, the intervention lowered younger children’s level of stress and scan issues to the level of older children, potentially lowering the threshold for awake scanning. Further research is needed to explore the role of child-friendly content in reducing the need for anesthesia in pediatric MRI.

## Conclusion

A child-friendly audio-visual intervention during MR imaging procedures delivers benefits for patient experience and workflow efficiency. The current study showed a reduction in stress levels, as well as fewer operational disruptions during an MRI exam for pediatric patients (aged 6–10 years).

## Supplementary Information

Below is the link to the electronic supplementary material.

Supplementary file1 (PDF 231 KB)

## Data Availability

No datasets were generated or analysed during the current study.
