# Supplementary material for: Magnetic resonance imaging related anxiety and workflow: impact of a child-friendly audio-visual intervention
Source: Pediatr Radiol. 2025 Jul 4;55(9):1934–42. doi: 10.1007/s00247-025-06308-0 (PMC12394259; doi:10.1007/s00247-025-06308-0)
Supplement: Supplementary file 1 — Supplementary file1 (PDF 231 KB) [file 247_2025_6308_MOESM1_ESM.pdf]

Supplementary materials

Part of article:

Magnetic resonance imaging related anxiety and clinical workflow: impact of a child-friendly audio-visual intervention

Journal: Pediatric Radiology

Methodology

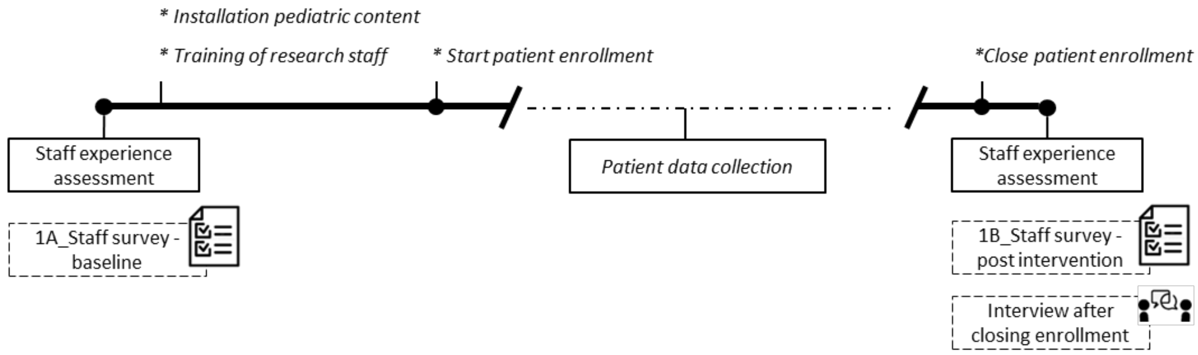

Fig. s1 Study overview on a timeline, including the data collection tools; baseline staff experience assessment prior to installation of the pediatric content and training of staff on research protocol (1A), and a post assessment after the patient data collection phase (1B)

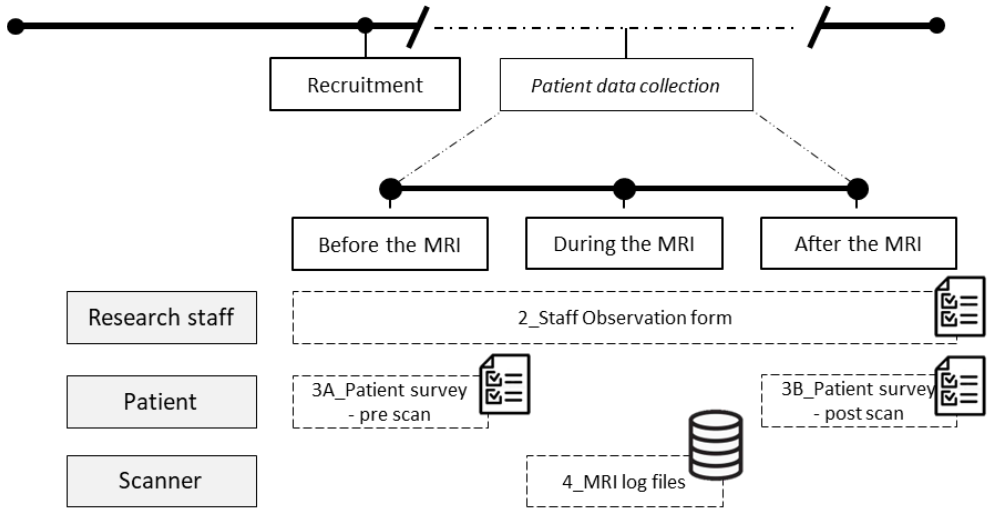

Fig. s2 Patient data collection on a timeline, including the tools; staff observation form throughout the patient's visit (2), the patient survey before the MRI (3A - before introduction of the intervention) and after the MRI (3B), and the MRI log files of the performed scan.

MRI magnetic resonance imaging

Table s1 Overview of all study measures

Staff observation form	Patient demographics
	Anxiety (modified yale preoperative anxiety scale [1-3])
	Patient demographics and scan characteristics
	Staff-rated patient stress (6-point Likert scale)
	Staff-rated image quality (6-point Likert scale)
	Workflow assessment: including ability to obtain diagnostic images (ordinal); image quality (6-point Likert); number of repeated scans, interventions, and pauses (numeric open-ended); estimated scan duration (6-point Likert scale)
Patient survey	Anxiety (Venham picture scale 1979)
	Self-rated stress (6-point visual analogue scale)
	Ease to lie still, comfort with the sound and duration of the MRI (6-point visual analogue scale)
	(only in intervention condition)
	Appreciation of the characters and clips (6-point visual analogue scale)

MRI magnetic resonance imaging

Results

Table s2 presents an overview of the number of participants in the three age groups. Given the unequal cell sizes and the overall small cell sizes in the youngest age groups, aggregation of the two younger age groups provides a more well-powered, robust analysis. Investigation of differences between the two age groups (6-8 and 8-10 years) showed no significant effects. In the main text of the paper, the data for the 6-8 years old and the 8-10 years old age groups are therefore aggregated into one larger age group (6-10 years old) (Table s3).

Table s2 Overview of number of participants in three age groups, per gender, per condition

	Control				Intervention			
	Boys	Girls	Other/ missing	Total	Boys	Girls	Other/ missing	Total
6-8 years	3	14	0	17	12	13	0	25
8-10 years	15	12	0	27	17	14	0	31
10+ years	23	22	1	46	14	14	1	29

Some groups are small, i.e. there were only 17 children in the youngest age group in the control condition with just 3 boys aged 6-8 years in this group. The data at the level of 6-8 and 8-10 years old should therefore be interpreted with caution

Table s3 Overview of number of participants in two age groups, per gender, per condition

	Control				Intervention			
	Boys	Girls	Other/ missing	Total	Boys	Girls	Other/ missing	Total
6-10 years	18	26	0	44	29	27	0	56
10+ years	23	22	1	46	14	14	1	29

Table s4 presents the staff-reported distress across the three age groups. To examine if merging the 6-8- and 8-10 years groups for the staff-reported distress is sensible, we conducted a 2 (condition: intervention vs. control) x 2 (age group: 6-8; 8-10) x 3 (timepoint: before MRI, during MRI, after MRI)

analysis of variance (ANOVA). There was no significant interaction between condition, age, and timepoint, F<1.

Table s4 Distress levels for the control and intervention condition, as reported by staff before, during, and after magnetic resonance imaging; analyses showing comparison of control versus intervention condition

	Patients: age 6-8 years				Patients: age 8-10 years			
	Control	Intervention		*Cohen's d*	Control	Intervention		*Cohen's d*
	M *(SD)*	M *(SD)*	*P*		M *(SD)*	M *(SD)*	*P*	
Before MRI	2.38 *(0.89)*	2.72 *(1.24)*	0.37	-	2.44 *(1.25)*	2.61 *(1.26)*	0.59	-
During MRI	2.81 *(1.05)*	2.52 *(1.16)*	0.38	-	2.33 *(1.29)*	2.16 *(0.90)*	0.53	-
After MRI	1.94 *(0.77)*	1.60 *(0.76)*	0.25	-	2.22[a] *(1.48)*	1.45[a] *(0.77)*	0.002	0.65

	Patients: age 10+ years			
	Control	Intervention		*Cohen's d*
	M *(SD)*	M *(SD)*	*P*	
Before MRI	2.18 *(1.17)*	2.45 *(1.18)*	0.34	-
During MRI	1.82 *(0.81)*	2.10 *(1.05)*	0.26	-
After MRI	1.51 *(0.76)*	1.66 *(0.81)*	0.51	-

M mean, *MRI* magnetic resonance imaging, *SD* standard deviation
[a]Significantly different at $P<0.05$

Table s5 presents the staff-reported scan issues across the three age groups. To examine if merging the 6-8- and 8-10 years groups for the staff-reported scan issues is sensible, we conducted a 2 (condition: intervention vs. control) x 2 (age group: 6-8; 8-10) ANOVA. There was no significant interaction between condition and age, $F<1$.

Table s5 Staff-reported scan issues index for the control and intervention condition

	Control		Intervention			
	M *(SD)*	*n*	M *(SD)*	*n*	*P*	Cohen's *d*
Age 6-8 years	0.50 *(0.21)*	*16*	0.37 *(0.16)*	*25*	0.02	0.68
Age 8-10 years	0.44 *(0.17)*	*27*	0.36 *(0.15)*	*31*	0.06	0.52
Age 10+ years	0.36 *(0.16)*	*45*	0.38 *(0.18)*	*29*	0.74	

M mean, *SD* standard deviation

Table s6 presents the logged scan issues across the three age groups. To examine if merging the 6-8- and 8-10 years groups for the logged scan issues is sensible, we conducted a 2 (condition: intervention vs. control) x 2 (age group: 6-8 or 8-10) analysis of covariance (ANCOVA) with number of sequences as covariate. There was no significant interaction between condition and age, F<1.

Table s6 Logged scans issues index for the control and intervention condition

	Control		Intervention			
	M *(SD)*	*n*	M *(SD)*	*n*	*P*	Cohen's *d*
Age 6-8 years	0.45 *(0.23)*	*16*	0.36 *(0.17)*	*24*	0.01	0.43
Age 8-10 years	0.44 *(0.12)*	*22*	0.37 *(0.17)*	*30*	0.07	0.47
Age 10+ years	0.39 *(0.17)*	*40*	0.43 *(0.20)*	*29*	0.35	

M mean, *SD* standard deviation

References

[1] Kain Z, Mayes L, Cicchetti D et al (1995) Measurement tool for preoperative anxiety in young children: The yale preoperative anxiety scale. Child Neuropsychology 1(3):203-210 https://doi.org/10.1080/09297049508400225

[2] Jenkins B, Fortier M, Kaplan SML, Kain Z (2014) Development of a Short Version of the Modified Yale Preoperative Anxiety Scale. Anesthesia Analgesia 119(3):643-650 https://doi.org/10.1213/ANE.0000000000000350

[3] Kain Z, Mayes L, Cicchetti D et al (1997) The Yale Preoperative Anxiety Scale: How Does It Compare with a "Gold Standard"? Anesthesia Analgesia 85:783-788 https://doi.org/10.1097/00000539-199710000-00012
